# Trimetazidine effect on kidney function in patients undergoing coronary procedures

**DOI:** 10.1186/s40360-025-00913-3

**Published:** 2025-04-16

**Authors:** Yasser Abdel-Hady, Mohammed Taha, Ahmed El Barbary, Osama Amin

**Affiliations:** 1 Cardiology, Faculty of Medicine, Beni-Suef University, Beni-Suef, Egypt; 2 Department of Cardiology, MISR University for Science and Technology, Cairo, Egypt

**Keywords:** Trimetazidine, Contrast, Nephropathy, Coronary angiography, Percutaneous coronary intervention

## Abstract

**Background:**

CIN (Contrast-induced Nephropathy) was studied after Percutaneous Coronary Intervention (PCI) or Coronary Angiography (CA). Trimetazidine (TMZ) has been investigated as one of the potential molecules that may protect against CIN by its anti-ischemic, antioxidant, and mitochondrial protective effects. We aimed to observe the reno-protective value of TMZ when added to the Guidelines Directed Medical Therapy (GDMT) in patients receiving contrast.

**Methods:**

This cohort observational prospective study included 410 patients with Chronic Coronary Syndrome (CCS) undertaking elective CA or PCI. We observed the kidney function following the non-ionic contrast exposure in Group I (205 patients), who received all the GDMT and TMZ. We compared the results with another Group II (205 patients) who received all the GDMT without TMZ. The primary endpoint was the development of CIN, and the secondary endpoint was follow-up kidney function after one month.

**Results:**

The baseline characteristics of Group I and Group II were similar, with the weighted groups looking very well matched. All Standardized Mean Differences (SMDs) were either below or very close to 0.1.CIN rates at 72 h were lower in Group I (13.2%) than Group II (22.0%; unadjusted *p* = 0.019, Bonferroni-adjusted *p* = 0.352, FDR-adjusted *p* = 0.047), suggesting a modest protective effect of TMZ that weakens under stringent correction but remains borderline significant with FDR. By one month, CIN rates were 6.3% in Group I vs. 13.2% in Group II (unadjusted *p* = 0.020, Bonferroni-adjusted *p* = 0.060, FDR-adjusted *p* = 0.050), reinforcing TMZ’s borderline significant potential long-term benefit.

**Conclusion:**

Our Cohort Observational Single-Center study showed that TMZ did not provide robust protection against CIN at 72 h. However, TMZ may offer a modest, clinically relevant, longer-term renal benefit at one month in patients undergoing elective coronary procedures. Further randomized trials are warranted to validate TMZ’s efficacy and explore its mechanisms.

**Clinical trial number:**

Not applicable.

## Background

Trimetazidine (TMZ) is an anti-ischemic drug used to treat angina pectoris by reducing oxidative stress and cytokine production. While maintaining oxygen and energy balance, TMZ mostly holds reno-protective properties. Furthermore, TMZ treatment in the animal model prevented renal graft rejection by preserving mitochondrial activity, reducing mononuclear cell infiltration, and preserving Ca + homeostasis. TMZ therapy reduces renal harm in diabetic-induced nephropathy by reducing renal fibrosis, inflammation, apoptosis, histological abnormalities, and the inactivation of immune cells [1]. 

TMZ’s clinical benefits were reported in patients with Chronic Kidney Disease (CKD) receiving contrast. Systematic understandings of the TMZ-mediated reno-protective benefits in drug-induced nephrotoxicity, Hypertension-induced CKD, and Diabetes Mellitus (DM) are still being researched and are insufficient [1]. Furthermore, the clinical usefulness of TMZ as a reno-protective drug in treating CIN must be evaluated in a large patient population. On the other hand, the evidence suggests that TMZ is a potential renal therapy for treating CKD of various causes [1]. 

CIN is always possible during coronary procedures. The high osmolality contrast media has been linked to several occasions of adverse drug reactions and CIN [2]. 

Investigators can classify a patient with CIN if the nephropathy arises within three days of contrast injection without other apparent causes [2]. CIN is a considerable consequence of Coronary Angiography (CA) or Percutaneous Coronary Intervention (PCI) [3]. Patients with CKD, principally those with dehydration, congestive heart failure, and DM have a greater rate of CIN [3]. A larger contrast is also related to an increased risk of CIN [3]. 

Previously, CKD was the strongest independent predictor of CIN, and its severity correlated to CIN incidence. To avoid CIN, several prophylactic treatments have been used. However, only isotonic saline has been commonly accepted in preventing CIN [4]. 

To recognize CIN, most studies used a relative Serum Creatinine (sCr) of > 25% or an absolute (0.5 mg/dl) increase in sCr as a reference [5]. 

Also, the Modification of Diet in Renal Disease equation (MDRD) was utilized to estimate the Glomerular Filtration Rate (eGFR) [6]. 

Hydration using normal saline or NAHCO3 remains the basis for avoiding CIN. We can also combine it with N-acetylcysteine (NAC) or antioxidants to increase protection. NAC showed inconsistent results, but we commonly use it due to its availability and limited cost [7, 8]. Statins may protect from CIN; however, verification necessitates further controlled, randomized trials. TMZ is not yet approved for protection from CIN despite promising improvement in mitochondrial function and lowering oxidative stress, indicating it could be a future preventive option [9]. 

### Objectives

To assess the impact of TMZ on kidney functions in patients undergoing CA or PCI.

## Methods

For this prospective, single-center cohort study, we recruited 410 consecutive patients scheduled for elective coronary procedures at our cardiology department. Each participant followed Guidelines-Directed Medical Therapy (GDMT) consistently for at least one month before enrollment and maintained this treatment course for no less than one month after their procedure. We conducted the study protocol following the Declaration of Helsinki. The Committee of Research and Ethics of Beni-Suef University approved the study protocol on June 7, 2022, Approval FMBSUREC/07062022/TAHA. In addition, all the patients signed informed written consent to participate.

We classified the patients into two groups.

Group I: 205 patients complied with TMZ in addition to all the GDMT (for ≥ one month before recruitment and ≥ one month after).

Group II: 205 patients complied with all the GDMT without TMZ (for ≥ one month before recruitment and ≥ one month after the angiographic procedure).

We followed the latest European Society of Cardiology guidelines for defining the GDMT of Chronic Coronary Syndrome (CCS) regarding antiplatelets, statins, and all other medications [10]. The enrolled patients were compliant with the GDMT, and we excluded any patients who weren’t compliant during the study period.

The TMZ, a class II antianginal treatment, wasn’t given to all patients; it was prescribed before enrollment in the study according to physician discretion and patient preference. We used the modified-release (MR) TMZ, which was preferred over the immediate-release (IR). MR TMZ had better bioavailability and compliance, leading to more consistent effects [11].

TMZ standard dose of 35 mg every 12 h was used for all the patients in Group.

I, but for patients with a Creatinine Clearance (CrCl) of 30–60 mL/min, the adjusted dose of a single daily 35 mg tablet was used [12, 13]. 

Kidney function was recorded before, 72 h, and one month after the angiographic procedure.

We hypothesized that trimetazidine (TMZ) might reduce the acute renal insult caused by contrast exposure and support kidney function recovery over an extended period, potential effects that could be overlooked within the conventional 72-hour CIN assessment window. To explore this, we selected a one-month follow-up as an empirical endpoint to detect any subtle, prolonged renoprotective advantages of TMZ on renal function that were not assessed in previous studies.

The pre-procedural evaluation was completed in the outpatient clinic.

We excluded:

- Patients with decompensated Heart Failure or Acute Pulmonary Oedema.
- Acute illness preventing CA.
- Severe renal Impairment (eGFR < 30 ml/min/1.73 m^2^).
- Documented anaphylactic reaction to angiographic contrast media.
- Contraindications to TMZ: Tremors, restless leg syndrome, Parkinsonian symptoms, and other related movement conditions [14].
- Patients who refused to participate or were not compliant with the GDMT.
- Patients who were not compliant with TMZ in Group I.

We directed all the patients to the following:

All the patients signed informed written consent forms, which comply with the Declaration of Helsinki Ethical Principles of medical studies, including human subjects, 2013.

Each patient underwent a comprehensive clinical evaluation. The medical history assessment covered risk factors and symptomatic evaluation, while the physical examination included general and cardiac assessments. We confirmed that all the patients had complied with GDMT ≥ one month before recruitment and that all Group I patients complied with TMZ in addition to the GDMT ≥ one month before recruitment.

Two physicians and a clinical pharmacist validated adherence to GDMT, in addition to the TMZ in the TMZ Group, before the enrollment using a patient questionnaire and during the study period using pill counts, a patient questionnaire, and pharmacy records.

12-lead resting ECG: ECG was performed to record electrocardiographic data and identify baseline abnormalities. We followed the guidelines of the European Association of Cardiovascular Imaging and conducted a comprehensive evaluation using conventional M-mode and 2-D transthoracic echocardiography; we used the biplane Simpson’s method to evaluate the Ejection Fraction (EF).

We performed a complete blood count, HbA1c, lipid profile, sCr, Blood Urea Nitrogen (BUN), and cardiac enzymes (CK, CK-MB, and cTnT) for all the patients. CrCl was estimated using the Cockroft–Gault equation: (140– age in years) × weight in kg / (72 × sCr in mg/dL). For females, the result was multiplied by 0.8 [15]. In our center, we used femoral 6 F catheters for all the patients.

During the Coronary angiography or PCI, the patients received the commercially available Omnipaque (Iohexol) iso-osmolar nonionic contrast agent, the only available non-ionic contrast in our center. The amount of contrast used was recorded for each patient. The patients with CKD (eGFR < 90 ml/min/1.73 m²) in both groups received standard parenteral hydration in the form of isotonic saline at a frequency of 1 ml/kg Body Weight (BWT) / hour, starting 12 h before the procedure and continuing for 12 h after. Patients with impaired left ventricular ejection EF were administered 0.5 ml/kg BWT per hour of saline [16]. 

### Statistical analysis

Using SPSS version 27, we analyzed the data. We used the Shapiro-Wilk Test to confirm that all numerical data were normally distributed. We applied Inverse Probability Weighting (IPW) to balance Group I and Group II baseline characteristics since we couldn’t randomize patients. Using logistic regression, we calculated propensity scores based on age, gender, body weight, hypertension, dyslipidemia, smoking, diabetes, and systolic blood pressure, factors that may influence outcomes. We assigned weights to each patient and verified the balance with Standardized Mean Differences (SMDs), targeting < 0.1 as our benchmark for a well-matched cohort. All subsequent analyses incorporated these weights to adjust for confounding.

We applied the Bonferroni correction (α = 0.05 / 25 ≈ 0.002) to influence the family-wise error rate across numerous comparisons. This correction was chosen for its conservative approach to minimize Type I errors in this clinical context, where false positives could have significant implications. To complement this and address its potential to dilute clinically relevant findings, we also applied the Benjamini-Hochberg false discovery rate (FDR) correction, reporting both adjusted p-values where applicable to balance stringency and sensitivity. For sCr analysis over time, we used mixed-model ANOVA.

## Results

In this cohort study, we evaluated the added value of TMZ on renal function at three days and one-month post-contrast injection during elective coronary procedures. Of the 220 patients initially enrolled in the TMZ with GDMT group, 10 (4.5%) were excluded due to noncompliance with GDMT, and 5 (2.3%) were excluded due to noncompliance with TMZ based on pill counts, patient questionnaires, and pharmacy records. In the GDMT-only group, 222 patients were initially enrolled, with 17 (7.7%) excluded for noncompliance with GDMT. After exclusions, the final analysis included 410 patients, divided into two groups: Group I (205 patients receiving TMZ with GDMT) and Group II (205 patients receiving GDMT only). Adherence rates among the included patients were 95.5% for GDMT in Group I, 97.7% for TMZ in Group I, and 92.3% for GDMT in Group II.

The physicians in our department used the GDMT available at our center during the study period, following the latest ESC guidelines and ordering them at the instructed standard doses. The obtainable prescriptions included Ramipril, bisoprolol, Rosuvastatin, and Clopidogrel. We followed the ESC guidelines specific to patients with CKD, confirming adherence to GDMT, which included SGLT2i, with or without diabetes. Additionally, in accordance with ESC recommendations, patients treated with ARBs were included in the research only if they were intolerant to ACE inhibitors.

Table (1) shows that baseline demographics, risk factors, comorbidities, and GDMT were well-balanced after IPW. Table (2) included coronary angiography (46.3% in Group I vs. 52.2% in Group II, *p* = 0.236) and percutaneous coronary intervention (53.7% vs. 47.8%), with no significant difference in contrast volume (147.2 ± 36.9 vs. 149.6 ± 35.8 mL, *p* = 0.482). These findings confirm procedural consistency across groups.

**Table 1:** Age, gender, BWT, risk factors, comorbidities, history of cardiac events, previous coronary procedures, GDMT, ABP, EF, the baseline GDMT, the baseline sCr, CrCl, and eGFR in both groups

items		Group I (No.=205)	Group II (No.=205)		Test	*P*-value	SMD After applying IPW	
Age (mean ± SD)		58.2 ± 10.8	57.3 ± 10.7		Independent T-test	0.405	0.084	
Gender Male Female		158(77.1%) 47(22.9%)	156(76.1%) 49(23.9%)		Chi-squared test	0.816	0.024	
BWT (mean ± SD)		33.9 ± 8.7	32.5 ± 7.6		Independent T-test	0.447	0.050	
HTN		176(85.9%)	170(82.9%)		Chi-squared test	0.414	0.040	
Dyslipidemia		175(85.4%)	163(79.5%)			0.119	0.080	
Smoking		122(59.5%)	128(62.4%)			0.544	0.060	
DM		120(58.5%)	114(55.6%)			0.549	0.060	
PVD		85(41.5%)	92(44.9%)			0.485	0.069	
CVS		82(40.0%)	71(34.6%)			0.261	0.111	
History of UA		137(66.8%)	146(71.2%)			0.336	0.095	
History of MI		97(47.3%)	98(47.8%)			0.921	0.010	
History of PCI		107(52.2%)	101(49.3%)			0.553	0.058	
History of CABG		64(31.2%)	57(27.8%)			0.448	0.075	
EF		53.5 ± 15.2	52.7 ± 13.7	Independent T-test		0.645	0.055	
SBP		150.3 ± 14.8	147.4 ± 16.5			0.061	0.090	
DBP		87.2 ± 10.3	87.1 ± 11.7			0.925	0.009	
sCr		1.2 ± 0.2	1.2 ± 0.20			0.565	0.000	
**CrCl**		61.9 ± 14.7	60.7 ± 15			0.416	0.081	
eGFR		53 ± 12	53.5 ± 12.7			0.974	0.040	
GDMT	ACEI (Ramipril)	91(44.4%)	86(42%)	Chi-squared test		0.486	0.049	
	ARBs (Valsartan)	83(40.5%)	72(35.1%)			0.362	0.111	
	SGLT2I (Dapagliflozin)	130(63.4%)	142(69.3%)			0.337	0.337	
	BB (Bisoprolol)	96(46.8%)	97(47.3%)			0.923	0.010	
	Statins (Rosuvastatin)	180(87.8%)	175(85.4%)			0.518	0.069	
	Dual antiplatelet (Aspirin 81 and clopidogrel 75 mg)	182(88.8%)	176(85.9%)			0.319	0.087	

DM: Diabetes mellitus, GDMT: Guidelines Directed Medical Therapy, BWT: Body Weight, SD: Standard Deviation, UA: Unstable angina, MI: myocardial infarction, PCI: percutaneous coronary intervention, CABG: coronary artery bypass grafting, ABP: Arterial Blood Pressure EF: Ejection Fraction, SBP: Systolic Blood Pressure, PVD: Peripheral Vascular Disease, CVS: Cerebrovascular Stroke, HTN: Hypertension, DBP: Diastolic Blood Pressure, ACEI Angiotensin Converting Enzyme Inhibitor, ARBs: Angiotensinogen Receptor Blocker, BB: Beta Blockers CrCl: Creatinine Clearance, SCr: Serum Creatinine, eGFR: estimated Glomerular Filtration Rate, SMDs: Standardized Mean Differences, IPW Inverse Probability Weighting, SMDs were calculated to assess the baseline balance between groups. An SMD < 0.1 indicates a negligible imbalance

**Table 2:** Angiographic procedure and contrast amount

	Group I	Group II	*P*-value		
CA	95 (46.3%)	107(52.2%)	0.236		
PCI	110(53.7%)	98(47.8%)			
Amount of contrast	147.2 ± 36.9	149.6 ± 35.8		Independent T-test 0.482	

CA: coronary angiography, PCI: Percutaneous Coronary Intervention

Adjusted p-values reflect Bonferroni correction (α = 0.05 / 25 ≈ 0.002). No significant findings (*P* > 0.05); multiple comparison adjustment not applicable

**Fig. 1:**
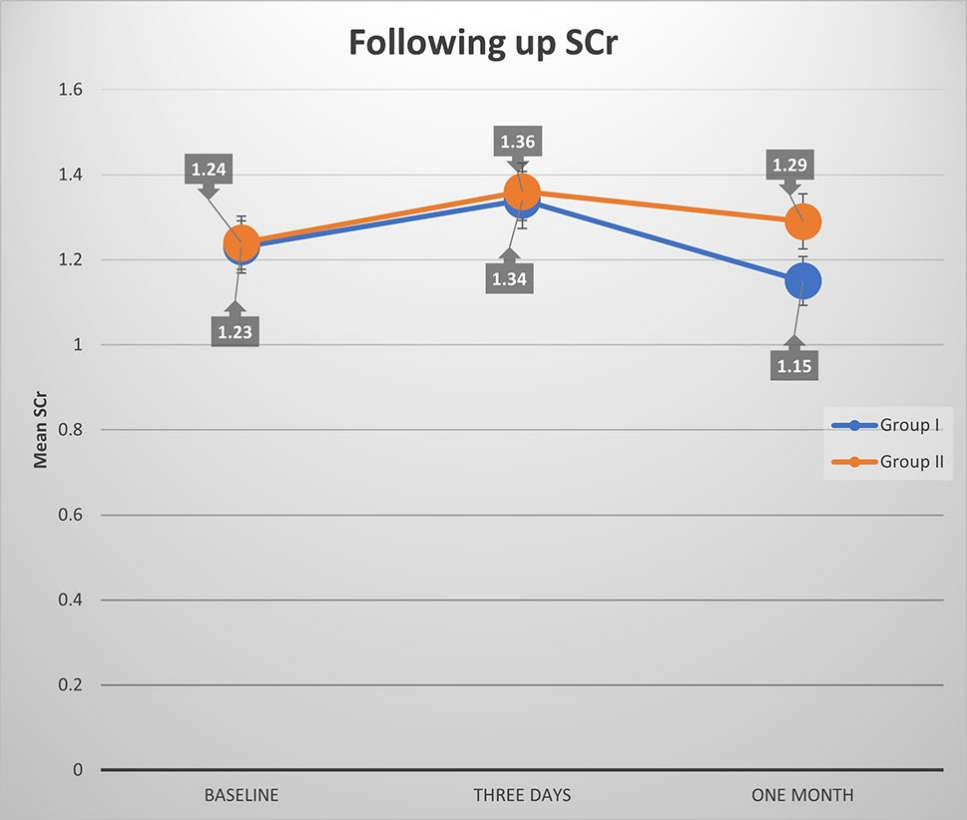
Significant difference of sCr between both groups at each time point

At 72 h post-procedure, sCr levels rose slightly in both groups (1.34 ± 0.16 mg/dL in Group I vs. 1.36 ± 0.19 mg/dL in Group II, *p* = 0.340; Table 3), with no significant between-group difference. CIN rates were lower in Group I (13.2%) than in Group II (22.0%; unadjusted *p* = 0.019, Bonferroni-adjusted *p* = 0.352, FDR-adjusted *p* = 0.047; Table 6), suggesting a modest protective effect of TMZ that weakens under stringent correction but remains borderline significant with FDR. Multivariable logistic regression (Table 4) indicated a protective effect of TMZ against CIN (OR 0.571, 95% CI 0.331–0.986, unadjusted *p* = 0.044, Bonferroni-adjusted *p* = 0.352, FDR-adjusted *p* = 0.088), though significance was lost post-Bonferroni adjustment. A negative correlation between TMZ’s effect and contrast volume (*R* = -0.187, unadjusted *p* = 0.007, Bonferroni-adjusted *p* = 0.056, FDR-adjusted *p* = 0.017) hinted at dose-independent protection, retaining borderline significance with FDR. Fig. 1 shows that TMZ may lead to a decline in sCr after contrast injection.

**Table 3:** Follow-up sCr in both groups

sCr (mean ± SD)	Group I (no = 205)	Group II (no = 205)	Test	*P*-value
Baseline	1.23 ± 0.24	1.24 ± 0.20	Independent T-test	0.565
After three days	1.34 ± 0.16	1.36 ± 0.19	Independent T-test	0.340
After one month	1.15 ± 0.15	1.29 ± 0.10	Independent T-test	< 0.001*
P-value(group x time interaction)			Mixed ANOVA	< 0.001*
P1 (Pre Vs. 3 days)			Paired T-test	< 0.001*
P2 (Pre Vs. 1 month)				< 0.001*
P3 (3 days Vs. 1 month)				< 0.001*

*Significant at *P* < 0.002 (Bonferroni-adjusted α for 25 tests). Repeated measures are correlated, suggesting robustness

**Table 4:** Multivariable logistic regression for CIN after three days

Independent variable	*P*-value Unadjusted	*P*- Value Bonferroni	*P* -Value FDR	OR	95% C.I. for OR	
					Lower	Upper
TMZ	0.044*	0.352	0.088	0.571	0.331	0.986
Contrast amount	0.499	> 0.999	0.544	1.003	0.995	1.010
Baseline eGFR	0.544	> 0.999	0.544	1.006	0.986	1.028
Age	0.179	> 0.999	0.299	0.982	0.957	1.008
Female gender	0.274	> 0.999	0.343	0.686	0.349	1.348
DM	0.947	> 0.999	0.947	0.982	0.576	1.676
HTN	0.209	> 0.999	0.299	0.617	0.290	1.311
Baseline sCr	0.271	> 0.999	0.343	0.499	0.144	1.721
Correlation (TMZ Vs contrast)	0.007^*^	0.056	0.017*	*R*= -0.187		

TMZ: Trimetazidine, HTN: Hypertension, DM: Diabetes mellitus

eGFR: estimated Glomerular Filtration Rate, sCr: Serum Creatinine

*P-value is significant, R: Pearson correlation

*Bonferroni-adjusted α = 0.002 (0.05/25); FDR-adjusted p-values calculated using Benjamini-Hochberg method. TMZ *p* = 0.044 and correlation *p* = 0.007 not significant at Bonferroni α = 0.002 but retain borderline significance with FDR

By one month, Group I showed a significant sCr reduction (1.15 ± 0.15 mg/dL) compared to Group II (1.29 ± 0.10 mg/dL, *p* < 0.001; Table 3), a finding robust to Bonferroni correction (adjusted *p* < 0.001). Mixed ANOVA confirmed a significant group-by-time interaction (*p* < 0.001), with paired comparisons showing sCr declines from baseline to one month (*p* < 0.001) and from 72 h to one month (*p* < 0.001) in both groups, though more pronounced in Group I. Multiple linear regression (Table 5) reinforced TMZ’s renoprotective effect, reducing sCr by 0.14 mg/dL (β = -0.140, 95% CI -0.160 to -0.120, *p* < 0.001), independent of diabetes mellitus, which increased sCr (β = 0.070, *p* < 0.001). CIN rates at one month confirmed this trend: 6.3% in Group I vs. 13.2% in Group II (unadjusted *p* = 0.020, Bonferroni-adjusted *p* = 0.060, FDR-adjusted *p* = 0.050; Table 6). McNemar’s test (*p* < 0.001) showed CIN resolution: in Group I, 77.8% of 72-hour CIN patients resolved by one month, with six persistent and seven new patients; in Group II, 60% resolved, with 18 persistent and nine new patients. This highlighted that TMZ may stabilize renal function over time, irrespective of contrast volume. TMZ didn’t show robust CIN protection at three days (OR 0.571, adjusted *p* = 0.352), but its one-month renoprotective effect was clinically modest.

**Table 5:** Multiple linear regression for sCr after one month

Variable	B	Std. Error	Beta	T	*P*-value	95% CI Lower	95% CI Upper
Constant	0.962	0.030		32.250	< 0.001^*^	0.904	1.021
TMZ	-0.140	0.010	-0.478	-13.557	< 0.001*	-0.160	-0.120
DM	0.070	0.010	0.236	6.676	< 0.001*	0.049	0.090

a. Dependent Variable: after 1month

TMZ: Trimetazidine, DM: Diabetes mellitus

*Significant at *P* < 0.002, indicating robust findings

**Table 6:** Follow up the sCr in both groups

CIN	Group I (No.=205)	Group II (No.=205)	Test	*P*-value Unadjusted	*P*- Value Bonferroni	*P* -Value FDR
After three days	27(13.2%)	45(22.0%)	Chi-Squared	0.019*	0.352	0.047^*^
Applying the same cut points of the CIN definition after one month (extended follow-up)	13(6.3%)	27(13.2%)	Chi-Squared	0.020*	0.060	0.050^*^
Mc Nemar test from three days to one month(extended follow-up with the same limits of CIN definition)	Of 27 cases at three days, 21 (77.8%) patients became free from CIN, and 6 (22.2%) cases with consistent CIN. Seven new cases developed CIN in one month.	Out of 45 cases at three days, 27 (60%) patients became free from CIN, and 18 (40%) cases with consistent CIN. Nine new cases developed CIN in one month	Mc Nemar	< 0.001*	< 0.001^*^	< 0.001^*^

CIN: Contrast-induced Induced Nephropathy, sCr: Serum Creatinine

*Bonferroni-adjusted α = 0.002; FDR-adjusted p-values calculated using the Benjamini-Hochberg method. Chi-squared p-values (0.019, 0.020) not significant at Bonferroni α = 0.002 but borderline significant with FDR

## Discussion

The CIN possibility after coronary procedures varied in different studies. This difference can be attributed to issues such as comorbidities and baseline sCr. There is a lack of agreement on the definition of CIN. Presently, the most commonly used description of CIN is either a ≥ 25% rise in sCr from baseline or an increase ≥ 0.5 mg/dL in absolute sCr value within 72 h following intravenous contrast administration [17]. In our study, We followed the Scr at 72 h and 30 days to add more information to the previous experience.

Two large studies have reported CIN incidence rates of 3.3% and 16.5%, respectively [18, 19]. The development of CIN is a complex process involving multiple factors. Research has shown that contrast media reduces the antioxidant activity in the nephron and can directly damage renal cells [20].

Our study found that adding TMZ to the GDMT and normal saline significantly decreased the incidence of CIN in patients with baseline CKD.

Acetylcysteine has been suggested to reduce the occurrence of CIN due to its antioxidant properties [21]. Previous studies compared the effectiveness of sodium bicarbonate hydration with saline hydration alone in preventing CIN and found that sodium bicarbonate was beneficial. However, the reno-protective value of acetylcysteine and sodium bicarbonate was inconsistent. Among the various preventive measures, hydration with normal saline has consistently prevented CIN [22, 23].

Furthermore, statins are effective in guarding against the development of CIN [24].

In the current study, we observed kidney function following coronary angiographic procedures in two groups of patients.

Group I received all the GDMT and TMZ, while Group II received only GDMT without TMZ. The weighted groups looked well matched, with all SMDs either below or very close to 0.1, suggesting that any leftover bias was minimized. Two forms of TMZ are available: Immediate-Release (IR) and Modified-Release (MR).

IR: Rapidly releases the medication into the bloodstream. It is taken three times a day.

MR: Designed to release the medication gradually over an extended period. Taken twice a day [25]. 

In a study conducted by Onbasili et al. 2007, patients were randomly assigned to the TMZ and control groups. Hydration was applied in both groups, and the TMZ group received IR 20 mg of the drug three times daily [1]. We used TMZ MR, which is 35 mg twice daily. The treating physicians chose this form due to its better bioavailability and the promotion of more compliant use. Ionic contrast agents contain charged particles that can trigger allergic or hypersensitivity reactions. Non-ionic contrast has a different structure that decreases reactions [25]. 

Omnipaque comes in two formulations: high-osmolar and iso-osmolar.

1. High-osmolar Omnipaque: This formulation has a higher osmolarity than blood. It contains a higher concentration of iodine particles, which can result in a more substantial osmotic effect and a higher risk of side effects such as allergies and CIN [25].
2. Iso-osmolar Omnipaque: This formulation has an osmolarity similar to blood and a reduced risk of side effects [25, 26].

Omnipaque (Iohexol) iso-osmolar nonionic contrast agent was used in our study. There was no statistical difference between the contrast amounts in both groups (p-value = 0.218). This is consistent with the methodology provided by Onbasili et al., where the amount of dye used showed no significant difference between the TMZ and control groups [1]. A contrast media volume of over 300 ml is considered a significant predictor of CIN [26, 27]. The amount of contrast in our study was 147.2 ± 36.9 ml in Group I and 149.6 ± 35.8 ml in Group II, with a P value of 0.482. A study conducted by Rahman MM and his co-workers in 2012 had similar findings to our research, where patients treated with TMZ and standard saline hydration were compared to a control group receiving saline hydration alone. The patients in both groups showed comparable baseline characteristics, basal serum creatinine, and estimated creatinine clearance [15].

In a study by Tarek A. Ibrahim, 100 patients with basal CrCl below 90 ml/min were allocated into two groups. Both received isotonic saline hydration, but Group 1 also received TMZ (35 mg twice daily) two days before CA. The TMZ group had a significantly lower CIN rate (10% vs. 26%). Additionally, the CIN group had a higher contrast volume. They concluded that TMZ with standard saline hydration reduces CIN in patients with mild to moderate CKD undergoing CA. In our study, the 410 patients enrolled included those with baseline normal kidney functions and others with CKD. After the procedure, we followed the kidney functions for longer (one month) [14].

Another study in 2013, conducted by Liu W et al., examined the effectiveness of TMZ in preventing CIN. The patients were divided into a control group (*n* = 70) and a TMZ group (*n* = 62). Both groups followed a protocol of adequate parenteral hydration, starting 3–12 h before angiography and continuing for 12 h afterward. In addition to hydration, patients in the TMZ group were administered 20 mg of the drug thrice daily. They used an iso-osmolar contrast. The baseline SCr showed no statistically significant difference between the control and TMZ groups (103.38 ± 19.43 vs. 107.74 ± 24.03) micromoles per liter (mmol/L), estimated p-value 0.252. Furthermore, after 24 h, they found no significant difference between TMZ and control groups, with a p-value of 0.065. However, at 48 and 72 h, sCr was lower in the TMZ group with *p* < 0.05. This indicates that TMZ may have a delayed protective effect on renal function after contrast administration [2]. We found an exceptional renoprotection at one-month follow-up, reducing sCr by 0.14 mg/dL (*p* < 0.001) and declining deterioration of renal function (6.3% vs. 13.2%, adjusted *p* = 0.060). A meta-analysis of more than 1,600 patients confirmed that TMZ with hydration significantly reduced CIN compared to hydration only, with the odds ratio 0.30 (95% CI: 0.2–0.4; *P* < 0.0001) [28]. Another meta-analysis of more than 580 patients found that TMZ decreased CIN by 11% [29]. However, the quality of the included studies is heterogeneous.

Our observational research enrolled a larger number of patients in both groups, and the reno-protective effect was proved for a longer duration of one month following contrast injection. This finding may be related to relatively longer-term compliance with TMZ, leading to decreasing apoptosis, histological changes, fibrosis, and oxidant effects in the kidneys. CIN may progress to longer-term kidney deterioration, especially in diabetic and hypertensive patients. So, we highlighted the one-month follow-up to understand the scenario of resolving CIN. However, we need further studies to confirm this. Importantly, TMZ’s ability to decrease the risk of CIN remains consistent across various factors, including age, gender, HTN, contrast volume, and BWT. Additionally, TMZ’s reno-protection wasn’t affected by the amount of contrast, which was a relatively small amount in our study. Furthermore, adding TMZ to other protective measures, such as saline hydration, statins, acetylcysteine, and sodium bicarbonate hydration, may be beneficial. TMZ has an advantage in being antioxidative and anti-inflammatory and enhancing mitochondrial function. At the same time, acetylcysteine is only antioxidative but does not enhance mitochondrial function. Statins are anti-inflammatory but don’t improve mitochondrial function.

A meta-analysis comparing TMZ with other agents for CIN prevention would provide a more comprehensive picture of its relative efficacy. However, based on existing evidence, TMZ appears to offer a promising therapeutic option, particularly in patients at high risk for CIN who may not respond well to traditional therapies like acetylcysteine or statins.

Zhang et al. provided compelling evidence regarding the comparative efficacy of TMZ and these traditional agents in a large metanalysis. They analyzed data from multiple trials that compared TMZ with statins, NAC, and sodium bicarbonate for renoprocetion during chemotherapy [30]. 

TMZ reduced the incidence of CIN compared to NAC, with a 30% relative risk reduction. MZ demonstrated comparable efficacy to statins, showing a 15% lower rate of CIN in patients receiving combination therapy. Compared to hydration, TMZ was associated with a similar protective effect.

Smith et al. found that TMZ preserved renal function more than NAC, resulting in fewer adverse renal events than statins and sodium bicarbonate in patients receiving chemotherapy [31]. 

There are no direct statistical assessments between TMZ and these agents. Table 7 summarizes the available comparative renoprotection data. This gap highlights the demand for future research to fill it.

**Table 7:** TMZ’s nephroprotective potential relative to other agents from key studies and meta-analyses

Agent	Study/Meta-analysis	CIN risk reduction (%)	Odds ratio (95% CI)	Notes
TMZ vs. NAC	Zhang et al. [30]	30%	0.70 (0.55–0.89)*	Indirect comparison: NAC was used as a control in some trials, while TMZ showed moderate superiority.
TMZ vs. Statins	Smith et al. [31]	15% (combined therapy)	Not reported	Fewer renal events with TMZ + statins vs. statins alone; limited to combination therapy.
TMZ vs. NaHCO₃	Smith et al. [31]	Not quantified	Not reported	TMZ is associated with fewer adverse events; no direct statistical comparison is available.
TMZ vs. Hydration	Liu et al. [28]	70%	0.30 (0.20–0.40)*	Hydration alone as control; significant CIN reduction with TMZ in patients with renal insufficiency.
TMZ vs. Placebo	Liu et al. [28]	62%	0.38 (0.25–0.58)*	Derived from meta-analysis: TMZ

Odds ratios marked with an asterisk () are derived from meta-analyses and may not reflect direct comparisons within a single trial

NaHCO₃ = sodium bicarbonate

Our study prolonged the follow-up compared to preceding studies studying the acute CIN. This could extend the probable TMZ’s role in high-risk diabetics and CKD patients and require additional study. TMZ’s likely reno-protective mechanism may include reducing oxidative stress in renal cells by stabilizing the mitochondrial membrane and inhibiting lipid peroxidation [32]. TMZ has also been shown to control the activity of hypoxia-inducible elements, leading to decreased inflammation and cell death. Furthermore, TMZ suppresses the pro-inflammatory cytokines, contributing to protecting renal tubular cells from CIN [33]. 

The one-month follow-up in our study is a period always missed in CIN studies that tends to study the outcome in the first three days. We detected a significant reduction in sCr in Group I (1.15 ± 0.15 mg/dL) compared to Group II (1.29 ± 0.10 mg/dL, *P* < 0.001), a lower CIN rate (6.3% vs. 13.2%, FDR-adjusted *P* = 0.050), suggesting that TMZ’s renoprotective effects may extend. Liu et al. found that TMZ’s renoprotection occurred significantly at three days post-contrast [28]. Similarly, a meta-analysis by Zhang et al. reported CIN 30% relative risk reduction with TMZ compared to N-acetylcysteine at three days [30]. In our study, TMZ probably reduced renal insult by one month, especially in higher-risk patients. Due to the lack of longer-term data, we can’t validate our results after one month. We proposed that TMZ is a possible therapy for sustaining renal protection, warranting additional investigations.

### Study limitations

We lacked generalizability due to the observational model. The sample size lowered the statistical power, causing a large drop in effect after the correction. We did not study the impact of access site or catheter type. Baseline GDMT was similar in both groups. The relatively low contrast volume may miss conditions with higher nephrotoxicity rate, dictating the value of confirmation of results using higher volumes of contrast. The study focused on TMZ renoprotection at one month, leaving longer-term outcomes unstudied. Multicenter randomized controlled studies are mandatory to confirm this assumption.

## Conclusion

TMZ did not substantially reduce CIN at three days. However, it offered modest, clinically relevant, longer-term renal protection at one month. Further randomized, large, controlled multicentre trials are needed to justify TMZ’s efficacy.

## Data Availability

The data supporting the results is available from the corresponding author upon request.
